# Can Exhaled Carbon Monoxide Be Used as a Marker of Exposure? A Cross-Sectional Study in Young Adults

**DOI:** 10.3390/ijerph182211893

**Published:** 2021-11-12

**Authors:** Ke-Ting Pan, Giovanni S. Leonardi, Marcella Ucci, Ben Croxford

**Affiliations:** 1 UCL Institute for Environmental Design and Engineering (IEDE), University College London, Gower Street, London WC1E 6BT, UK; ke-ting.pan.16@ucl.ac.uk (K.-T.P.); m.ucci@ucl.ac.uk (M.U.); 2 Graduate Institute of Aerospace and Undersea Medicine, National Defense Medical Centre, Taipei 114, Taiwan; 3 Radiation, Chemical and Environmental Hazards, UK Health Security Agency, Didcot OX11 0RQ, UK; giovanni.leonardi@phe.gov.uk; 4 Department of Public Health, Environments and Society, London School of Hygiene and Tropical Medicine, London WC1H 9SH, UK

**Keywords:** carbon monoxide, CO half-life, CO elimination, cigarette, smoking

## Abstract

Carbon monoxide (CO) poisoning is a major public health issue worldwide. People are exposed to CO in their daily lives, with one of the common sources of CO being cigarette smoking. Inhalation of CO leads to elevated carboxyhaemoglobin (COHb) levels in the blood and also in exhaled CO concentration. Several factors have been shown to affect COHb concentration and COHb half-life. However, factors affecting exhaled CO concentration and exhaled CO half-life are not well understood. The present study aimed to investigate the potential factors related to baseline exhaled CO concentration and exhaled CO half-life among smokers. A cross-sectional study was conducted between 26 January and 30 June 2019, and young adults were recruited into the study. A total of 74 participants (mean age: 27.1 years, 71.6% males and 28.4% females) attended the study. They were invited to complete a questionnaire, including demographic, physiological, and behavioural factors. Then, exhaled CO measurements were taken. These measurements were taken before and after smoking a single cigarette for smokers and only once for non-smokers. The average baseline exhaled CO concentration was 6.9 ± 4.9 ppm for smokers and 1.9 ± 0.5 ppm for non-smokers. The mean of exhaled CO half-life was around 273.3 min (4.6 h) for smokers. No difference was seen in exhaled CO half-life between light smokers and heavy smokers in the smoking group. Gender and cigarettes smoked weekly affected baseline exhaled CO in smokers. Even though height seemed to positively associate with exhaled CO half-life, the relationship disappeared when adjusting by gender and weight. Therefore, exhaled CO could be used as a marker of CO exposure, but we cannot ignore the factors mentioned in the study. For future study, considering factors related to smoking habits and smoking style are recommended as these may affect total inhaled CO.

## 1. Introduction

Carbon monoxide (CO) is an odourless, tasteless, colourless, and poisonous gas produced from the incomplete combustion of organic compounds [1,2]. In many countries, CO was the leading cause of the fatal poisonings reported [3]. It behaves similarly to oxygen in the body, but has around 200–260 times higher affinity to haemoglobin (Hb) and forms as carboxyhaemoglobin (COHb) in the blood [2,4]. Exposure to high amounts of CO may result in hypoxia and produce a series of adverse health effects, such as headaches, nausea, fatigue, respiratory dysfunction, tissue damage and even death [1,5,6]. In the United States, there were a total of 24,890 CO poisoning deaths (including unintentional and intentional) from 1999 to 2014 (annual death rate of 0.5/100,000) [7]. In the WHO European Region report, CO-related deaths were recorded at a total of 140,490 between 1980 to 2008 (annual death rate of 2.2/100,000) [8].

The treatment guide for CO poisoning is to help patients to eliminate CO as soon as possible. The COHb half-life has been estimated as approximately 4 h in room air [5,9] and approximately 30 min with Hyperbaric oxygen (HBO) therapy [9]. Several factors have been shown to affect COHb elimination, such as severity and duration of exposure to CO, ventilation rate, age, gender, and blood volume [10,11,12,13]. However, the effects of cigarette smoking on CO uptake and elimination remains controversial [14,15,16]. In an observational study of a CO poisoning incident in a public high school, Burney et al. investigated the factors related to COHb half-life and found cigarette smoking did not impact COHb half-life [14]. However, Cronenberger et al.’s study showed that smokers have a longer COHb half-life than non-smokers [16].

Smoking prevalence varies by country, ranging from 43.4% in Greece to 14.7% in Iceland from Our World in Data [17]. It is the major source of CO exposure. For smokers, smoking exposes people to a high concentration of CO [18]. In the WHO report, the CO concentration in tobacco smoke is around 4.5% (45,000 ppm), and smokers inhale air with a concentration of about 400–500 CO ppm during smoking [19]. Therefore, smokers usually have a higher concentration of COHb in the blood, around 6% to 9% of COHb, compared to 1% to 3% of COHb in non-smokers [20,21]. Exhaled CO concentration has been shown to be highly correlated with COHb concentration, especially in healthy smokers [21,22,23]. The use of devices to monitor CO in breath has increased in research settings and clinics to diagnose CO exposure [24,25,26]. Generally, without potential air pollution, the exhaled CO concentration would be expected in a range of 1–4 ppm in non-smokers and 2–18 ppm in smokers [24]. Suppose the exhaled CO concentration of the participants and patients was higher than expected, in that case, they might be exposed to CO. Breath CO monitors have provided a non-invasive, relatively low-cost and quicker way to measure CO concentrations compared to the blood COHb test.

However, factors affecting exhaled CO as a marker of CO exposure are not well characterised. Even though Jarvis et al. reported that exhaled CO measurement could distinguish smokers from non-smokers, they mentioned that a few smokers could not be identified due to not inhaling the smoke very deeply [27]. In 2020, Ghorbani et al. indicated that breath sampling may also have an impact on exhaled CO concentration [28]. Moreover, Chatrchaiwiwatana and Ratanasiri stated that the cut-off point of differentiating exhaled CO concentration between smokers and non-smokers might be affected by age [29]. Therefore, factors affecting the exhaled CO concentration and exhaled CO half-life are worth exploring and addressing. The poor quantitative characterisation of the effect of demographic, physiological factors, and smoking behaviour on exhaled CO limits its value for modelling CO exposure and documenting its health effects.

In the present study, breath CO monitors were used to measure CO concentration from the participants. The primary aim of this study was to explore the factors, including demographic, physiological and behavioural factors, and smoking status, that affect baseline exhaled CO concentration and exhaled CO half-life.

## 2. Materials and Methods

### 2.1. Study Design and Participant Recruitment

The present study was a cross-sectional study conducted between 26 January and 30 June 2019. The participants were recruited through physical posters placed at University College London (UCL) and Goodenough College. The participants were young, healthy, aged 18 to 34 years old, university students or their friends, with no pregnancy and no history of illness related to lung function changes. Participants were categorised as “smokers” if they had smoked more than 100 cigarettes through their entire life till the present [30,31]. “Light smokers” were defined as those who smoked less than ten cigarettes per day, and “heavy smokers” were those who smoked equal to or more than ten cigarettes per day [32,33]. In the study, the sample size was calculated using data from a previous study [24]. The sample size was calculated using STATA software by setting 80% for the power and 0.05 for the significance value. As a result, the researcher estimated that at least 13 participants were needed for each group, including smokers (light smokers and heavy smokers) and non-smokers. This study was approved by the UCL Research Ethics Committee (REC) (Project ID: 14201/001).

### 2.2. Data Collection Procedure

On the day participants attended the study, non-smokers were excluded if they had smoked before attendance (n = 1), and smokers if they could not properly follow the protocol of exposure measurement (n = 9). The study protocol contained two parts, including questionnaires and exposure measurements. After recruitment, participants were invited to fill out the consent and questionnaire. The questionnaire included age, gender, height, weight, BMI, ethnicity, diet, menstrual cycle and smoking habits, such as years of smoking, type of cigarettes, number of cigarettes smoked daily and weekly and time since the last cigarette. Participants were also asked if they had exercise or had been exposed to CO (ex. Exposure to secondhand smoke, gas fire, cars exhaust, etc.) before attendance for the study measurements, and their responses were recorded.

### 2.3. Exposure Measurement

In the exposure measurement part, baseline exhaled CO concentration was measured in all participants. After their baseline exhaled CO concentration had been recorded, smokers were asked to smoke one control cigarette with the same brand and type (Seven Stars, Japan Tobacco, Tokyo, Japan). Then, the researcher (K.-T.P.) measured exhaled CO concentration immediately after smoking and at 30 min, 60 min, 90 min and 120 min after smoking. Moreover, smokers were asked not to smoke for at least four hours before attending the study [34,35]. This period of four hours was based on the half-life of COHb in people breathing natural air [5], aiming to minimise the effects of the last cigarette. The researcher recorded the time since the last cigarette before the exhaled CO test of each participant.

The exhaled CO half-life was calculated from the formula below. The method was described by Weaver et al. and Ozturan et al. [15,36]. In the equation, if concentration 1 (c1) and concentration 2 (c2) are the levels of exhaled CO concentration taken at time 1 (t1) and time 2 (t2) during CO ‘wash-out’ time, then the half-life of exhaled CO can be calculated. The exhaled CO half-life is also calculated as follows:

$$\mathrm{CO}\;\mathrm{half}-\mathrm{life}\;=\left(t2-t1\right)\times \ln\left(2\right)/\left[\ln\left(c1/c2\right)\right]$$

- t1 is time point 1
- t2 is time point 2
- c1 is the concentration of exhaled CO in t1
- c2 is the concentration of exhaled CO in t2.

Exhaled CO concentration was monitored by a breath CO monitor, the ‘Micro^+^™ Smokerlyzer^®^’ (Bedfont Scientific Ltd., Medical manufacturer, Maidstone, UK). The participants were asked to hold their breath for 20 s and then blow continuously and slowly into the Smokerlyzer mouthpiece, following the procedure described in the manual of Smokerlyzer. The researcher stayed with the participants and instructed them about the protocol at the time of their attendance for the study.

### 2.4. Statistical Analysis

Analyses were conducted using Microsoft Excel, IBM SPSS Statistics 26 (IBM, Armonk, NY, USA) and Stata IC 15 (TX: StataCorp LLC, College Station, TX, USA). Descriptive statistics were computed and reported as mean ± standard deviation (SD) for age, gender, height, weight, BMI and exhaled CO at each time point. Univariable analysis was then conducted to describe the relationship of each variable with baseline exhaled CO concentration and exhaled CO half-life. Mean differences between the two groups, such as gender and smoking status, were compared by the Student’s t-test. If variables had more than two groups, such as ethnicity, analysis of variance (ANOVA) was performed to understand the difference across each group. When the number of participants was less than 10, the nonparametric Mann–Whitney U test or the Kruskal-Wallis H test was applied to compare median values. The chi-square test was applied when analyzing the relationship by gender, ethnicity, smoking status, etc. (categorical variable data). A Pearson’s correlation was used to study the relationship between baseline exhaled CO concentration and age, height, weight, etc. (two quantitative and continuous variables). A backward stepwise multivariable regression was then applied to investigate the factors related to baseline exhaled CO concentration and the exhaled CO half-life. A standardised beta coefficient was used to rank the most important variables in the stepwise multivariable regression model presented. A *p*-value of <0.05 was considered to be statistically significant, and all *p*-values were given for two-sided tests.

## 3. Results

A total of 84 participants were recruited for the study. After exclusion, exhaled CO concentrations were assessed for 74 participants, including 48 smokers (28 light smokers and 20 heavy smokers) and 26 non-smokers.

Table 1, part (A) displays the basic demographics of the study participants. The mean age was 27.1 ± 4.0 with a mean height of 173.0 ± 9.3 and weight of 69.1 ± 13.5. Twenty-one participants were female, and the majority of ethnicities were Asian or White/Caucasian in both smokers and non-smokers. Around 30% of the participants were exposed to CO or exercised before attending the study. When comparing the characteristics between smokers and non-smokers, smokers had a higher concentration of baseline exhaled CO than non-smokers (6.9 ± 4.9 vs. 1.9 ± 0.5, *p*-value < 0.001), and a higher mean of weight and BMI. Also, compared to non-smokers, there was a higher percentage of males among smokers, and more smokers exercised before attending the study.

Table 1, part (B) describes the demographics and smoking-related characteristics between light smokers and heavy smokers. The baseline exhaled CO was 4.8 ± 2.6 ppm in light smokers and 10.0 ± 5.8 ppm in heavy smokers (*p*-value < 0.001). Light smokers had fewer cigarettes smoked daily and weekly compared to heavy smokers. A higher percentage of males were in the heavy smokers’ group than light smokers (95.0% vs. 71.4%, *p*-value = 0.039). The majority of ethnicities were Asian or White/Caucasian with a similar distribution of light smokers and heavy smokers (*p*-value = 0.304). Other factors, such as age, height, weight, BMI, years of smoking, time since the last cigarette, puffs, smoking duration, ethnicity, and type of cigarettes used to smoke, showed no significant difference between light smokers and heavy smokers.

**Figure 1:**
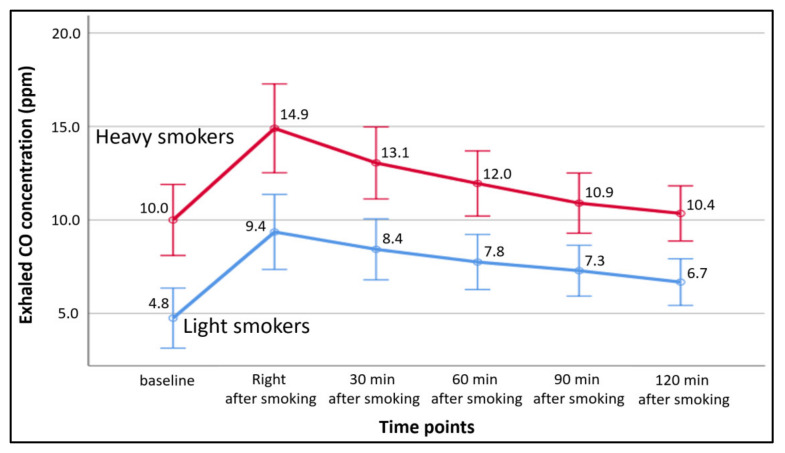
Exhaled CO concentration for light smokers and heavy smokers at different time points. Error bar—means ± 95% CI (Confidence Interval).

Figure 1 presents the exhaled CO concentration for light smokers and heavy smokers at different time points. The average exhaled CO concentrations changed following the same pattern in both smoking groups (light smokers and heavy smokers) through the time points, and heavy smokers had a higher exhaled CO concentration than light smokers at all time points.

Table 2 reports that baseline exhaled CO concentration and exhaled CO half-life showed a significant difference between males and females. In contrast, the exhaled CO half-life showed no significant difference between light smokers and heavy smokers. The average exhaled CO half-life among the smokers was 273.3 ± 95.6 min (4.6 ± 1.6 h).

Table 3, part (A) indicates that there was a moderate relationship between cigarettes smoked daily (r = 0.394, *p*-value = 0.006)/ weekly (r = 0.417, *p*-value = 0.003) and the baseline exhaled CO concentration, which means the number of cigarettes smoked daily/weekly was positively associated with the concentration of baseline exhaled CO. Table 3, part (B) shows a weak relationship between height and exhaled CO half-life (r = 0.357, *p*-value = 0.016), indicating that height was positively associated with exhaled CO half-life.

Table 4 and Table 5 show the factors that affect the baseline exhaled CO concentration and exhaled CO half-life of smokers. The final models only included significant and borderline significant factors. The results showed that gender (β = −5.491, *p*-value = 0.020) and cigarettes smoked weekly (β = 0.051, *p*-value = 0.004) affect the baseline exhaled CO concentration. Height and age showed borderline significance. If a person was older or smoked more cigarettes weekly, the baseline CO concentration increased. Height affects the time of exhaled CO half-life (β = 4.878, *p*-value = 0.007). If a person was taller, the exhaled CO half-life time increased. However, once the results were adjusted by gender and weight, the impact of height disappeared. Gender, height and weight did not affect the exhaled CO half-life in the regression analysis.

## 4. Discussion

To date, non-invasive monitors for CO assessment have been widely used. This study is the first study to use a breath CO monitor to calculate exhaled CO half-life and explore factors affecting baseline exhaled CO concentration and exhaled CO half-life. Our results using exhaled CO were relatively similar to those from studies using COHb from blood as an exposure marker, where half-life is about 4–5 h [2,5]. The average age of the participants was 27 years old since the inclusion criteria were 18–34 years old. Therefore, the potential effects of ageing of the lungs were eliminated [37,38]. In the study, the difference of baseline exhaled CO concentration between smokers and non-smokers was around 5 ppm (6.9 ppm vs. 1.9 ppm), which was similar to the data from Kozienice in Maga et al.’s study, in which the average baseline exhaled CO concentration was 6.5 ppm in smokers and 1.1 ppm in non-smokers [24]. However, the baseline exhaled CO concentration was less than the study by Maga et al., based in Krakow (smokers vs. non-smokers, 12.3 ppm vs. 7.0 ppm) and Warsaw (smokers vs. non-smokers, 14.4 ppm vs. 5.1 ppm) [24]. Another study also showed a higher baseline exhaled CO concentration than our study, and the mean exhaled CO concentration was 3.6 ppm for non-smokers and 17.1 ppm for smokers [26]. The lower baseline CO concentration in the study may be related to the lower number of heavy smokers, lower background CO concentration, shorter years of smoking, and the mean of time since the last cigarette, which was much longer than other studies [24,26].

The baseline CO concentration of the smokers was between 1 ppm to 24 ppm. It showed that some of the smokers’ baseline exhaled CO concentration was similar to non-smokers, which was around 1.9 ppm. The possible reason for the low exhaled CO baseline concentration in smokers might be the long period since the last cigarette. In our study, the average time since the last cigarette was around 23 h. The COHb half-life for a healthy person breathing air is approximately 4 h [5]. If a person stops smoking for a sufficiently long period, the exhaled CO concentration could be similar to non-smokers. Besides, some studies reported that smokers could lower their CO exposure by reducing the puff volume, the puffs smoked and the tendency and depth of inhaling [18,39,40,41,42]. In terms of puffs, males generally tended to have a higher puff volume, a longer puff duration and shorter intervals between puffs than females [42]. Above all, these may be highly related to smoking habits and hard to control. Therefore, this might be a reason for the big variation of exhaled CO concentration within and between different studies [20,24,26,43]. Even though the exposure of CO from smoking may be highly affect by smoking habits and hard to control, smoking is the major source of CO exposure in the population. Future studies should consider the possible ways to measure the actual amount of CO that goes into the body while smoking.

Moreover, some studies showed that cigarettes themselves might play a role in CO exposure in smoking, such as paper porosity, filter, cigarette CO level, cigarette nicotine level and type of cigarettes [18,40,44]. Laugesen et al.’s study reported that even though the increased CO ppm was similar in hand-rolled cigarettes and factory-made cigarettes, the CO ppm increase per g of tobacco burnt was higher in hand-rolled cigarettes than in factory-made cigarettes [44]. Therefore, the cigarettes in the present study were controlled to being the same brand and type to avoid the effects of the properties of different cigarettes.

In the regression model, gender and cigarettes smoked weekly affected baseline exhaled CO concentration. The gender effect may be due to more heavy smokers in the male group, as heavy smokers tend to have a higher concentration of COHb [3,24,45]. Moreover, some studies showed that females may have lower exhaled CO concentrations during menstruation due to loss of blood, which has a high affinity with CO [46]. The baseline exhaled CO concentration was positively associated with the number of cigarettes smoked daily and weekly, similar to other studies [20,24,26,39,43]. Some studies also reported that exhaled CO concentration is higher for participants who smoke and inhale more deeply [39,43]. In our study, the concentration of exhaled CO showed no difference before and after smoking in a few participants. Some of them claimed that they did not inhale the smoke into their lungs, while some of the participants said they did inhale deeply. The same situation was also found in Jarvis et al.’s study [27].

The average COHb half-life in smokers was 4.5 h in our study, similar to other studies [2,5]. Light smokers and heavy smokers showed no significance in exhaled CO half-life. Similar findings were also demonstrated in the studies [14,15]. However, Cronenberger et al. (2008) have reported the median (range) COHb half-life was 30.9 h (7.13–367) in adult smokers [16], which was longer compared with the results from exhaled CO half-life in our study (median, 4.1 h). The possible reason that COHb half-life was longer in Cronenberger et al.’s study than in the present study might be the younger age of participants in the present study (age range: 18–34) compared to the participants in Cronenberger et al.’s study (age range: 21–63). Moreover, even though some studies showed that cigarette smoking might affect lung function and reduce gas exchange efficiency [47,48], the effects may be reduced due to only young and healthy participants being recruited.

Moreover, there were only 45 participants in the regression. The reason was that in three participants, the exhaled CO concentration did not decrease after 120 min after smoking. Therefore, their exhaled CO half-life could not be calculated. Besides the equipment error for the three participants, the reason for exhaled CO concentration without decreasing after 120 min after smoking might be the longer exhaled CO half-life of smokers than non-smokers [16]. Therefore, it is hard to detect the decrease of exhaled CO concentration within 120 min.

Gender and height showed their effects on exhaled CO half-life in the correlation and univariable test. Height was also found to have a positive association with exhaled CO half-life in smokers in multivariable regression. However, when controlling for gender and weight (significant and borderline significant factors in the univariable test), height, gender and weight together showed no significant effects on exhaled CO half-life in the regression model. Gender has been postulated to affect COHb half-life in studies [11,49]. Female smokers had a shorter exhaled CO half-life compared to male smokers, which may be due to females having a lower Hb mass and higher alveolar ventilation than males [11,49]. Some studies have suggested that alveolar ventilation and total Hb mass, more than gender, may play a critical role in COHb elimination and half-life [11,12,13]. Besides gender and height, weight showed a slightly positive association with exhaled CO half-life with a borderline significance (Table 3, part (B)). Generally, heavier people have increased blood volume and have a longer COHb half-life [12,13].

Study limitations. Firstly, the participants smoked a controlled cigarette in their usual manner. The number of puffs, interval time between puffs and the depth of smoking were hard to control and may affect exhaled CO concentration. Fortunately, the puffs and smoking duration were recorded, and the researcher recruited more participants than estimated in each group to reduce the effects of the big variation in exhaled CO concentration on the analysis. Moreover, different CO exposure methods could be used in future studies, such as the DL_CO_ test and CO-rebreathing experiment, which are safer and utilise a known dose of CO exposure under clinical and medical staff control. Secondly, many females tended to reject the study and were not willing to report their smoking status when recruiting participants. This situation resulted in there being more males than females involved in the study. Also, the lower number of female participants makes it hard to see if the menstrual cycle would affect the exhaled CO concentration and exhaled CO half-life. Thirdly, the backward stepwise regression was applied to find the factors affecting baseline CO concentration and exhaled CO half-life. However, this method was only based on statistical results without evidence from the literature. Different approaches could be considered in the future. Fourthly, breath CO monitors are most used for healthy participants due to the protocol of breath-holding for 20 s might be hard to perform for patients with certain conditions, such as lung illness and chest pain. Finally, the participants smoked outdoors due to the smoking regulations at the university and did the exhaled CO experiment indoors. Even though there may be a delay after smoking to the exhaled CO measurement, the exact times recorded in the study were much less than the exhaled CO half-life. Therefore, this time delay is not expected to affect the study significantly.

## 5. Conclusions

This is the first study to calculate exhaled CO half-life using a breath CO monitor and showed relatively similar results compared to the COHb half-life measured in blood, especially in young healthy adults. Therefore, exhaled CO could be used as a marker of CO exposure. For example, patients presenting with an exhaled CO concentration suggest CO exposure above what is expected in smokers, pointing to the need to search for CO sources of exposure different from smoking. However, some factors, such as gender and cigarettes smoked weekly, might influence the value of exhaled CO as a marker of exposure. Those factors should be considered when interpreting the results. Further research should consider additional factors related to smoking habits, such as type/brand of cigarettes, interval time between puffs and the depth of smoking. Moreover, the effect of the menstrual cycle, alveolar ventilation and total Hb mass on exhaled CO concentration and COHb half-life could be explored in the future.

## Figures and Tables

**Table 1:** (**A**)**.** Demographics, physiological and baseline exhaled CO of the study participants by smoking status. (**B**)**.** Demographics, physiological, smoking-related and baseline exhaled CO characteristics of light smokers and heavy smokers.

**(A)**				
**Characteristics**	**Total**	**Smokers**	**Non-Smokers**	***p*-Value**
	**(n = 74)**	**(n = 48)**	**(n = 26)**	
Age (years)	27.1 ± 4.0	26.6 ± 4.5	27.9 ± 2.7	0.202
Height (cm)	173.0 ± 9.3	174.3 ± 8.1	170.6 ± 10.9	0.100
Weight (kg)	69.1 ± 13.5	72.1 ± 13.8	63.2 ± 11.1	0.007 **
BMI (kg/m^2^)	23.1 ± 3.3	23.6 ± 3.6	21.8 ± 2.3	0.026 *
Baseline exhaled CO (ppm)	5.2 ± 4.6	6.9 ± 4.9	1.9 ± 0.5	<0.001 **
Gender				0.013 *
Male	53 (71.6)	39 (81.3)	14 (53.9)	
Female	21 (28.4)	9 (18.7)	12 (46.2)	
Ethnicity				0.507
Asian	45 (60.8)	27 (56.3)	18 (69.2)	
Black/Africa American	2 (2.7)	1 (2.1)	1 (3.9)	
Hispanic/Latino	4 (5.4)	2 (4.2)	2 (7.7)	
White/Caucasian	21 (28.4)	16 (33.3)	5 (19.2)	
Mixed Ethnicity	2 (2.7)	2 (4.2)	0 (0)	
Exposure CO before the study				0.199
None	53 (71.6)	32 (66.7)	21 (80.8)	
Yes	21 (28.4)	16 (33.3)	5 (19.2)	
Exercise before study				0.047 *
None	52 (70.3)	30 (62.5)	22 (84.6)	
Yes	22 (29.7)	18 (37.5)	4 (15.38)	
(**B**)				
**Characteristics**		**Light Smokers**	**Heavy Smokers**	***p*-Value**
		**(n = 28)**	**(n = 20)**	
Age (years)		27.2 ± 4.4	25.9 ± 4.6	0.302
Height (cm)		173.4 ± 8.8	175.5 ± 7.0	0.386
Weight (kg)		70.9 ± 11.2	73.9 ± 16.8	0.456
BMI (kg/m^2^)		27.2 ± 4.4	25.9 ± 4.6	0.302
Baseline exhaled CO (ppm)		4.8 ± 2.6	10.0 ± 5.8	<0.001 **
Years of smoking (year)		8.6 ± 4.7	9.0 ± 5.0	0.783
Time since last cigarette (hour ago)		34.3 ± 69.4	7.6 ± 3.7	0.093
Cigarettes smoked (daily)		3.2 ± 2.0	12.6 ± 4.0	<0.001 **
Cigarettes smoked (weekly)		23.1 ± 16.6	89.6 ± 28.6	<0.001 **
Puffs		12.4 ± 4.3	11.3 ± 3.9	0.368
Smoking duration (min)		3.6 ± 0.8	3.3 ± 1.3	0.250
Gender				0.039 *
Male		20 (71.4)	19 (95.0)	
Female		8 (28.6)	1 (5.0)	
Ethnicity				0.304
Asian		14 (50.0)	13 (65.0)	
Black/Africa American		0 (0)	1 (5.0)	
Hispanic/Latino		2 (7.1)	0 (0)	
White/Caucasian		10 (35.7)	6 (30.0)	
Mixed Ethnicity		2 (7.1)	0 (0)	
Exposure CO before the study				0.301
None		17 (60.7)	15 (75.0)	
Yes		11 (39.3)	5 (25.0)	
Exercise before study				0.762
None		18 (64.3)	12 (60.0)	
Yes		10 (35.7)	8 (40.0)	
Type of cigarette				0.883
Factory-made cigarette		19 (67.9)	14 (70.0)	
Hand-rolled cigarette		7 (25.0)	4 (20.0)	
Both		2 (7.1)	2 (10.0)	

Data are reported as the mean ± standard deviation or number (percentage). Where a significant difference between groups was found, the *p*-values are highlighted: * *p*-value < 0.05; ** *p*-value < 0.01.

**Table 2:** (**A**). Comparison of baseline exhaled CO concentration between different groups in smokers. (**B**). Comparison of exhaled CO half-life between different groups in smokers.

**(A)**		
**Variable (n = 48)**	**Baseline Exhaled CO (ppm)**	***p*-Value**
	**Mean ± SD ^1^**	
Total (n = 48)	5.2 ± 4.6	
Gender		0.002 **
Male (n = 39)	7.7 ± 5.1	
Female (n = 9)	3.6 ± 2.1	
Smoking status		<0.001 **
Light smokers (n = 28)	4.8 ± 2.6	
Heavy smokers (n = 20)	10.0 ± 5.8	
Ethnicity		0.264
Asian (n = 27)	7.9 ± 5.9	
Black/African-American (n = 1)	9	
Hispanic/Latino (n = 2)	5.0 ± 4.2	
White/Caucasian (n = 16)	5.9 ± 3.0	
Mixed ethnicity (n = 2)	2.5 ± 0.7	
Type of cigarette		0.744
Factory-made cigarette (n = 33)	7.3 ± 1.0	
Hand-rolled cigarette (n = 11)	5.7 ± 0.8	
Both (n = 4)	7.0 ± 1.4	
Exposure to CO before the study		0.094
None (n = 32)	7.8 ± 1.0	
Yes (n = 16)	5.3 ± 0.7	
Exercise before study		0.586
None (n = 30)	6.6 ± 0.8	
Yes (n = 18)	7.4 ± 1.4	
(**B**)		
**Variable (n = 45)**	**CO Half-Life (Minutes)**	***p*-Value**
	**Mean ± SD ^1^**	
Total (n = 45)	273.3 ± 95.6	
Gender		0.010 *
Male (n = 36)	288.1 ± 96.1	
Female (n = 9)	213.9 ± 70.4	
Smoking status		0.396
Light smokers (n = 25)	262.3 ± 90.5	
Heavy smokers (n = 20)	287.0 ± 22.9	
Ethnicity		0.462
Asian (n = 25)	282.8 ± 101.8	
Black/African-American (n = 1)	314.4	
Hispanic/Latino (n = 2)	205.8 ± 52.8	
White/Caucasian (n = 15)	272.7 ± 95.4	
Mixed Ethnicity (n = 2)	206.1 ± 35.6	
Type of cigarette		0.848
Factory-made cigarette (n = 31)	272.3 ± 93.6	
Hand-rolled cigarette (n = 10)	280.1 ± 123.2	
Both (n = 4)	264.5 ± 27.1	
Exposure CO before the study		0.281
None (n = 29)	284.8 ± 106.5	
Yes (n = 16)	252.4 ± 70.2	
Exercise before the study		0.486
None (n = 29)	280.8 ± 94.4	
Yes (n = 16)	259.7 ± 99.3	

^1^ SD—standard deviation. Where a significant difference between groups was found, the *p*-values are highlighted: * *p*-value <0.05; ** *p*-value <0.01.

**Table 3:** (**A**). Correlation of baseline exhaled CO concentration with demographics, physiological and smoking habits in smokers. (**B**). Correlation of exhaled CO half-life with demographics, physiological and smoking habits in smokers.

**(A)**		
**Variable**	**Correlation Coefficient**	***p*-Value**
Age (years)	0.163	0.267
Height (cm)	0.061	0.681
Weight (kg)	0.136	0.356
BMI (kg/m^2^)	0.132	0.373
Years of smoking (year)	−0.089	0.553
Time since last cigarette (hour ago)	−0.269	0.067
Cigarettes smoked (daily)	0.394	0.006 **
Cigarettes smoked (weekly)	0.417	0.003 **
Puffs	−0.239	0.101
Smoking duration (min)	−0.130	0.379
(**B**)		
**Variable**	**Correlation Coefficient**	***p*-Value**
Age (years)	0.007	0.965
Height (cm)	0.357	0.016 *
Weight (kg)	0.292	0.051
BMI (kg/m^2^)	0.159	0.297
Years of smoking (year)	0.051	0.714
Time since last cigarette (hour ago)	0.032	0.835
Cigarettes smoked (daily)	0.033	0.828
Cigarettes smoked (weekly)	−0.062	0.688
Puffs	−0.199	0.189
Smoking duration (min)	0.025	0.872

Where a significant correlation was found, the *p*-values are highlighted: * *p*-value < 0.05; ** *p*-value < 0.01.

**Table 4:** Factors affecting baseline CO concentration in smokers.

Variable ^1^ (n = 47)	R^2^ = 0.349, Adjusted R^2^ = 0.287			
	β ^2^	Beta ^3^	95% CI ^4^	*p*-Value
Gender (female/ male)	−5.491	−0.439	(−10.071, −0.911)	0.020
Cigarettes smoked (weekly)	0.051	0.407	(0.017, 0.084)	0.004
Height (cm)	−0.193	−0.310	(−0.417, 0.030)	0.088
Age (year)	0.287	0.260	(−0.0001, 0.573)	0.050

^1^ Variables included when running backwards stepwise regression: age, gender, height, weight, BMI, exposure CO, exercise, type of cigarette, cigarettes smoked weekly, years of smoking, time since the last cigarette, number of puffs and smoking duration, ^2^ β—un-standardised coefficient, ^3^ Beta–standardised coefficient, ^4^ 95% CI—95% Confidence Interval.

**Table 5:** (**A**). Factors affecting exhaled CO half-life in smokers. (**B**). Factors affecting exhaled CO half-life for smokers.

**(A)**				
**Variable ^1^ (n = 45)**	**R^2^ = 0.163, Adjusted R^2^ = 0.143**			
	**β ^2^**	**Beta ^3^**	**95% CI ^4^**	***p*-Value**
Height (cm)	4.878	0.403	(1.431, 8.326)	0.007
(**B**)				
**Variable (n = 45)**	**R^2^ = 0.141, adjusted R^2^ = 0.078**			
	**β ^1^**	**Beta ^2^**	**95% CI ^3^**	***p*-Value**
Height (cm)	2.483	0.209	(−3.141, 8.109)	0.378
Gender (female/male)	−26.893	−0.114	(−125.814, 72.028)	0.586
Weight	0.718	0.106	(−1.837, 3.273)	0.573

(**A**) ^1^ Variables included when running backwards stepwise regression: age, gender, height, weight, BMI, exposure CO, exercise, type of cigarette, cigarettes smoked weekly, years of smoking, time since the last cigarette, number of puffs and smoking duration, ^2^ β—un-standardised coefficient, ^3^ Beta—standardised coefficient, ^4^ 95% CI—95% Confidence Interval. (**B**) ^1^ β—un-standardised coefficient, ^2^ Beta—standardised coefficient, ^3^ 95% CI—95% Confidence Interval.

## Data Availability

All the data is presented in the article.
